# In Memoriam

**Published:** 1884-10

**Authors:** 


### IN MEMORIAM.

### ACTION OF THE BOARD OF HEALTH IN REFERENCE TO THE DEATH OF DR. DRAKE.

The following is an extract from the minutes of the Board of Health of the city of Atlanta, September 13, 1884:

Whereas, in the unerring wisdom of the Supreme Ruler of the universe, our friend and colleague, Dr. William G. Drake, has been removed from our midst by death; and

Whereas, by his decease the board of health has lost a safe and wise counsellor, the city a faithful and conscientious officer, and society a genial and true member; therefore, be it

Resolved, That, while we bow in reverent submission to the divine decree, we are profoundly sensible of the absence of our brother from his accustomed place and deeply deplore his loss.

Resolved, That the sincere sympathy of this Board be most respectfully tendered the sadly stricken family, and that a page in our records be inscribed to his memory.

Resolved, That these resolutions be published in the city papers, in the ATLANTA MEDICAL AND SURGICAL JOURNAL and in the Southern Medical Record.

W. S. ARMSTRONG, M. D.,
*President.*
JAMES B. BAIRD, M. D.
*Secretary.*
FRANK P. RICE,
AARON HAAS.

